# OAT3 Participates in Drug–Drug Interaction between Bentysrepinine and Entecavir through Interactions with M8—A Metabolite of Bentysrepinine—In Rats and Humans In Vitro

**DOI:** 10.3390/molecules28041995

**Published:** 2023-02-20

**Authors:** Aijie Zhang, Fanlong Yang, Yang Yuan, Cai Li, Xiaokui Huo, Jing Liu, Shenzhi Zhou, Wei Li, Na Zhang, Jianfeng Liu, Shiqi Dong, Huirong Fan, Ying Peng, Jiang Zheng

**Affiliations:** 1 Wuya College of Innovation, Shenyang Pharmaceutical University, Shenyang 110016, China; 2 State Key Laboratory of Functions and Applications of Medicinal Plants, Key Laboratory of Pharmaceutics of Guizhou Province, Guizhou Medical University, Guiyang 550025, China; 3 Key Laboratory of Radiopharmacokinetics for Innovative Drugs, Chinese Academy of Medical Sciences, and Institute of Radiation Medicine, Chinese Academy of Medical Sciences and Peking Union Medical College, Tianjin 300192, China; 4 School of Chinese Materia Medica, Tianjin University of Traditional Chinese Medicine, Tianjin 301617, China; 5 College of Pharmacy, Dalian Medical University, Dalian 116044, China; 6 Heping District Center for Disease Control and Prevention, Tianjin 300070, China

**Keywords:** Y101, entecavir, renal excretion, organic anion transporters, drug-drug interaction

## Abstract

Bentysrepinine (Y101) is a novel phenylalanine dipeptide for the treatment of hepatitis B virus. Renal excretion played an important role in the elimination of Y101 and its metabolites, M8 and M9, in healthy Chinese subjects, although the molecular mechanisms of renal excretion and potential drug–drug interactions (DDIs) remain unclear. The present study aimed to determine the organic anion transporters (OATs) involved in the renal disposition of Y101 and to predict the potential DDI between Y101 and entecavir, the first-line agent against HBV and a substrate of OAT1/3. Pharmacokinetic studies and uptake assays using rat kidney slices, as well as hOAT1/3-HEK293 cells, were performed to evaluate potential DDI. The co-administration of probenecid (an inhibitor of OATs) significantly increased the plasma concentrations and area under the plasma concentration–time curves of M8 and M9 but not Y101, while reduced renal clearance and the cumulative urinary excretion of M8 were observed in rats. The time course of Y101 and M8 uptake via rat kidney slices was temperature-dependent. Moreover, the uptake of M8 was inhibited significantly by probenecid and benzylpenicillin, but not by *p*-aminohippurate or tetraethyl ammonium. M8 was found to be a substrate of hOAT3, but Y101 is not a substrate of either hOAT1 or hOAT3. Additionally, the entecavir inhibited the uptake of M8 in the hOAT3-transfected cells and rat kidney slices in vitro. Interestingly, no significant changes were observed in the pharmacokinetic parameters of Y101, M8 or entecavir, regardless of intravenous or oral co-administration of Y101 and entecavir in rats. In conclusion, M8 is a substrate of OAT3 in rats and humans. Furthermore, M8 also mediates the DDI between Y101 and entecavir in vitro, mediated by OAT3. We speculate that it would be safe to use Y101 with entecavir in clinical practice. Our results provide useful information with which to predict the DDIs between Y101 and other drugs that act as substrates of OAT3.

## 1. Introduction

**Scheme 1:**
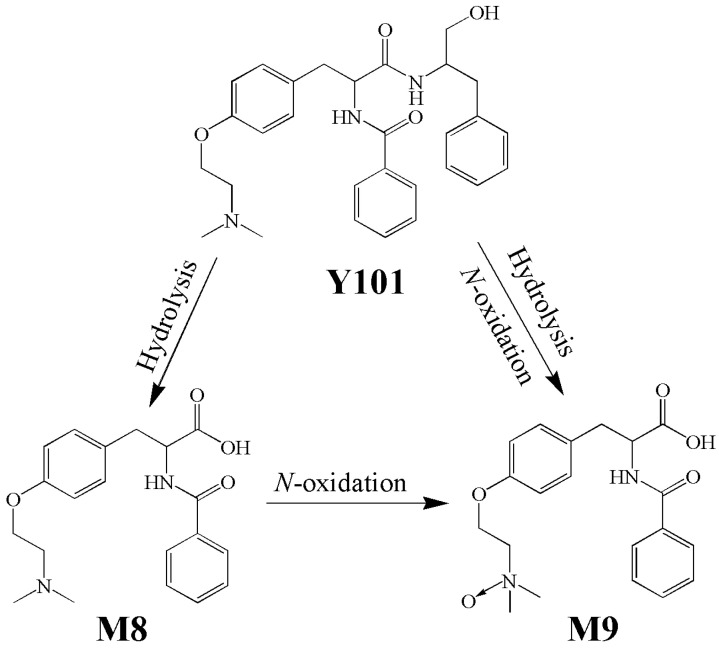
Proposed biotransformation pathways of Y101 in humans.

Bentysrepinine (*N*-[*N*-benzoyl-O-(2-dimethylaminoethyl)-l-tyrosyl]-l-phenylalaninol hydrochloride, Y101), a novel phenylalanine dipeptide currently under clinical development [1], was designed for the treatment of hepatitis B virus (HBV) infection. Previous pharmacological studies reported that Y101 exhibited potent inhibitory effects on HBsAg and HBV-DNA replication in a dose-dependent manner through binding with HBV DNA polymerase and had significant activity against lamivudine and entecavir-resistant HBV replication in A64 cells [1,2,3,4]. Moreover, Y101 also has revealed protective effects on concanavalin A and alpha-naphthylisothiocyanate-induced liver injury [5,6]. Recently, the results of phase I studies indicated that Y101 exhibited acceptable safety and tolerability in healthy Chinese volunteers [1,7]. This clinical study reported that Y101 was extensively metabolized in humans in vivo, as the exposures to hydrolytic metabolite M8 and hydrolytic/oxidative metabolite M9 (Scheme 1) were much higher than that of the parent drug, although M8 and M9 did not show any pharmacological activity in a molecular docking study [8]. The total cumulative renal excretion of Y101, M8 and M9 was more than 30%, suggesting that urinary excretion was an important elimination pathway. The finding was consistent with some other peptidomimetic drugs such as JBP485, β-lactam antibiotics, bestatin and angiotensin-converting enzyme inhibitors (ACEIs), which are substrates of organic anion transporter 1 (OAT1) and OAT3 in the kidneys [9,10,11,12]. However, the mechanisms involved in the renal excretion of Y101, M8 and M9 remain unclear.

The current standard of therapy and first-line agent against HBV in clinical practice is entecavir (ETV) [13]. The cytochrome P450 enzymes in the liver are reportedly not involved in the disposition of ETV, and the predominant elimination route of ETV is via the kidneys in unchanged form [14]. Several studies have reported that ETV is a substrate of OAT1, OAT3 and organic cation transporters 2 (OCT2) in OAT1, OAT3 and OCT2-transfected cells [15,16]. Furthermore, it was reported that OATs and OCT2 were involved in the drug–drug interactions (DDIs) between ETV and JBP485 [15] or crizotinib [17], respectively. For a variety of therapeutic goals, ETV may be combined with diverse antivirals or other anti-HBV drugs in clinical practice [18].

Organic anion transporters are mainly distributed in the basolateral membrane of the proximal tubules in the kidneys, which exert considerable influence on the renal elimination of anionic xenobiotics and drugs, such as *p*-aminohippurate (PAH), benzylpenicillin (PCG), estrone-3-sulfate (ES), probenecid (PRO), antiviral drugs, and β-lactam antibiotics [12]. Moreover, Y101 and its related metabolites are also potentially substrates of OATs. Therefore, an OAT-dependent DDI between Y101 and ETV could potentially occur when the two drugs are simultaneously administered.

With these observations in mind, this study aimed to (1) demonstrate which transporters are involved in the renal disposition of Y101 and its metabolites using in vivo and in vitro models, including in vivo pharmacokinetics, in vitro rat kidney slices, and in vitro hOAT1 and hOAT3-HEK293 cells; and (2) validate whether Y101 and ETV interact with each other following co-administration in vivo, as well as elucidating its molecular pharmacokinetic mechanisms via a transporter-mediated DDI. The results provide useful information with which to elucidate the mechanisms of the renal disposition of Y101 and its metabolites and to help to predict the DDIs mediated by transporters.

## 2. Results

### 2.1. Effect of PRO on Pharmacokinetics of Y101, M8 and M9 in Rats

**Figure 1:**
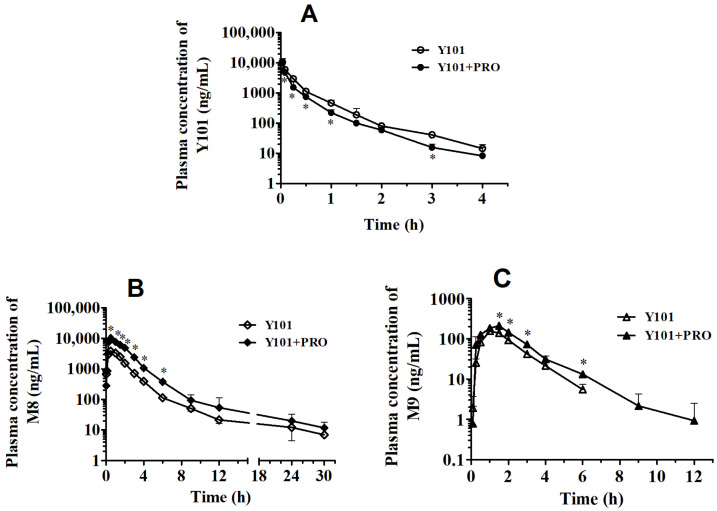
Mean plasma concentration–time curves of Y101 (**A**), M8 (**B**) and M9 (**C**) after intravenous administration of Y101 (25 mg/kg) or Y101 + PRO (100 mg/kg). Data are expressed as mean ± SD (* *p* < 0.05 vs. Y101 group; *n* = 3).

An inhibitor of OATs, PRO is often used when testing drugs in vivo to verify the potential renal excretion mechanism via transporters. To investigate the effect of PRO on the pharmacokinetics of Y101, M8 and M9, the time-course changes of plasma were determined in rats co-administered (i.v.) with Y101 and PRO. A slightly increased plasma clearance (CL_P_) rate was observed in the rats treated with Y101 + PRO relative to that of the animals treated with Y101 alone (Table 1; CL_P_, from 8.94 ± 1.65 L/h/kg to 12.4 ± 1.01 L/h/kg), while the AUC and half-life (*t*_1/2_) of Y101 remained almost unchanged (Table 1). The plasma concentrations and AUCs of the M8 and M9 increased significantly in comparison with those of the control group (Figure 1B,C, Table 1). Additionally, 2.6- and 1.4-fold increases in *C*_max_ values for M8 and M9 were observed, along with 163% and 56% elevations in the AUC values.

**Figure 2:**
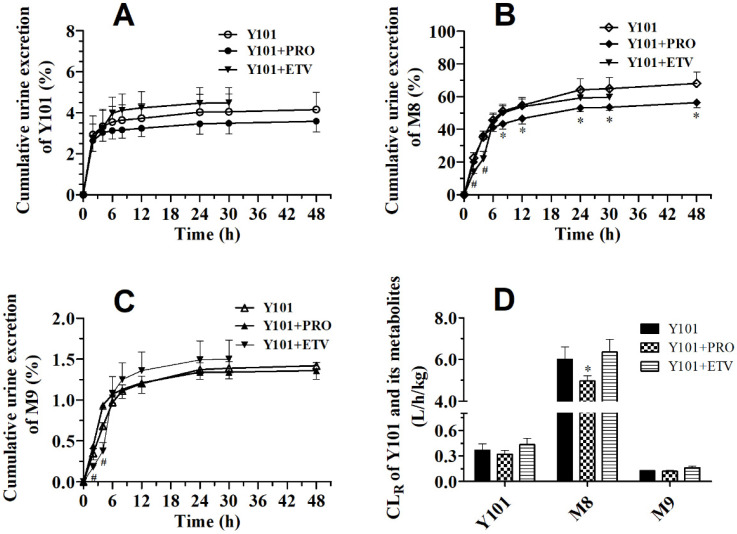
Cumulative urine-excretion curves of Y101 (**A**), M8 (**B**) and M9 (**C**) and the renal clearances of Y101 and its metabolites (**D**) after intravenous administration of Y101 (25 mg/kg), Y101 + PRO (100 mg/kg) and Y101 + ETV (0.06 mg/kg). Data are expressed as mean ± SD (* *p* < 0.05 vs. Y101 group; *n* = 6).

To investigate the DDI taking place in the kidneys, we examined the cumulative urinary excretion of Y101, M8 and M9 in the presence or absence of PRO in the rats. Following the intravenous administration of Y101, the cumulative urinary excretion of Y101, M8 and M9 at 48 h post-dose were 4.16 ± 0.843%, 68.1 ± 6.97% and 1.42 ± 0.04% of the dose, respectively (Figure 2). After the co-administration of Y101 and PRO, the cumulative urinary excretion of Y101, M8 and M9 at intervals of 0 to 48 h were 3.59 ± 0.526%, 56.3 ± 3.00% and 1.36 ± 0.106%, respectively (Figure 2). The cumulative excretion of the M8 in the urine was inhibited significantly (*p* < 0.05) by the PRO. Moreover, the CL_R_ of the M8 decreased significantly when the Y101 was co-administered with the PRO (Table 1). These results indicate that the PRO decreased the renal elimination of the M8 from the plasma.

### 2.2. Pharmacokinetic DDI between Y101 and ETV in Rats

**Figure 3:**
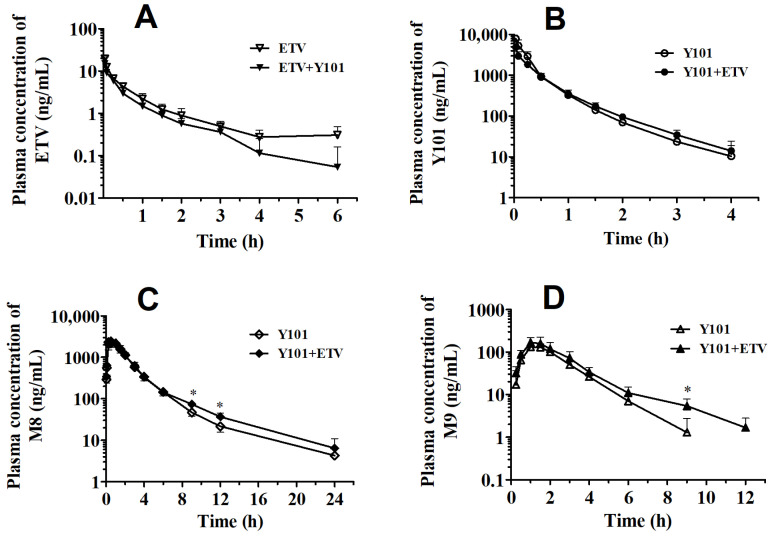
Mean plasma concentration–time curves of ETV (**A**), Y101 (**B**), M8 (**C**) and M9 (**D**) after intravenous administration of Y101 (25 mg/kg) and Y101 + ETV (0.06 mg/kg). Data are expressed as mean ± SD (* *p* < 0.05 vs. Y101 group; *n* = 4).

**Figure 4:**
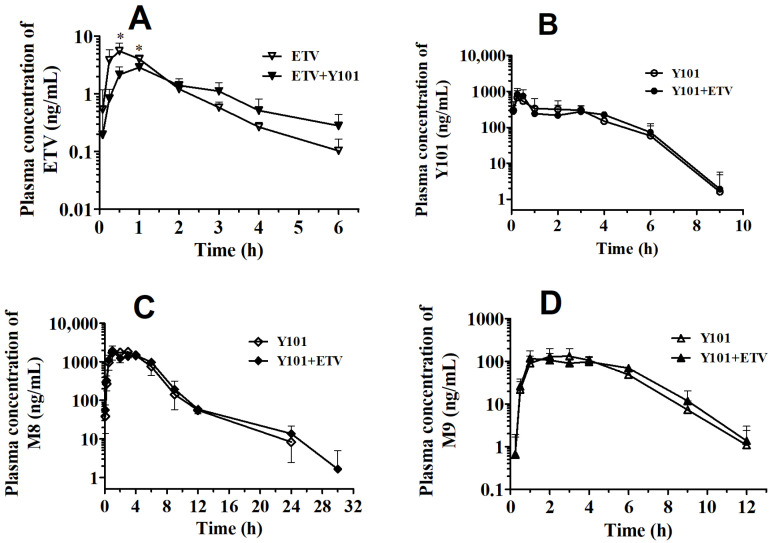
Mean plasma concentration–time curves of ETV (**A**), Y101 (**B**), M8 (**C**) and M9 (**D**) after oral administration of Y101 (60 mg/kg) and Y101 + ETV (0.06 mg/kg). Data are expressed as mean ± SD (* *p* < 0.05 vs. Y101 group; *n* = 4).

ETV is a commonly prescribed antiviral drug in clinical practice. Considering patients often receiving a couple of antiviral drugs for therapy, we examined the DDI between the ETV and the Y101 in vivo. Therefore, Y101 and ETV were co-administered intravenously and orally in rats. The concentration–time curves and pharmacokinetic parameters of the Y101, M8 and M9 are shown in Figure 3 and Figure 4, Table 2 and Table 3. Compared with the control group, the CL_p_ of the ETV and the *t*_1/2_ of the M8, the M9 slightly increased, while no significant changes in the other pharmacokinetic parameters were observed, following the co-administration of Y101 and ETV intravenously to rats (Figure 3 and Table 2). Interestingly, we did not find significant alterations in any of the pharmacokinetic parameters between Y101 alone and the Y101 + ETV groups when oral administration was applied to animals instead of intravenous treatment (Figure 4 and Table 3).

**Figure 5:**
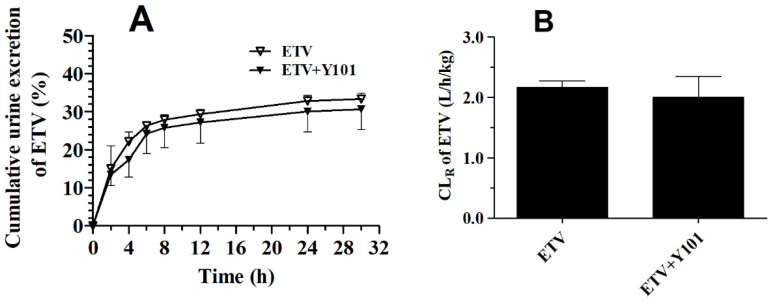
Cumulative urine-excretion curves of ETV (**A**) and the renal clearance of ETV (**B**) after intravenous administration of Y101 (25 mg/kg) and Y101 + ETV (0.06 mg/kg). Data are expressed as mean ± SD (* *p* < 0.05 vs. ETV group; *n* = 6).

To examine the alteration in the renal excretion of the two drugs, the ratio of the cumulative excretion of ETV in urine in the rats after intravenous administration was found to be 33.4 ± 1.55% at 30 h post-dose (Figure 5). Moreover, the co-administration of ETV and Y101 failed to change the cumulative urinary excretion of ETV (Figure 5). Following the intravenous administration of Y101 + ETV, the cumulative excretion rates of Y101, M8 and M9 in the urine at 30 h post-dose were 4.48 ± 0.742%, 59.7 ± 5.78% and 1.50 ± 0.232% of the dose, respectively (Figure 2). No significant differences were found in the urinary excretion of ETV, Y101, M8 or M9 for the Y101 + ETV group. The values of CL_R_ for Y101, M8, M9 and ETV also remained unchanged in the Y101 + ETV group (Table 2).

### 2.3. Effects of OAT Substrates, Inhibitors and ETV on the Uptake of Y101 or M8 in Rat Kidney Slices

**Figure 6:**
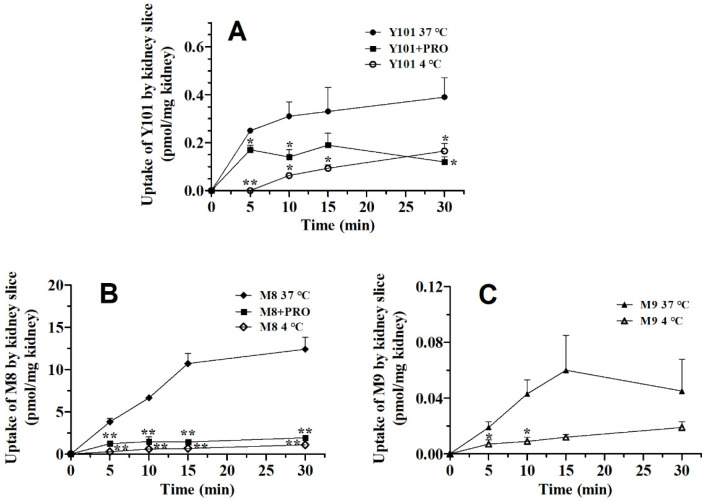
Time-dependent and temperature-dependent uptake of Y101 (2.0 µM, (**A**)), M8 (5.0 µM, (**B**)) and M9 (1.0 µM, (**C**)) in kidney slices. The concentration of PRO was 100 µM. (*, *p* ˂ 0.05; **, *p* ˂ 0.01). Data are expressed as mean ± SD, *n* = 3.

**Figure 7:**
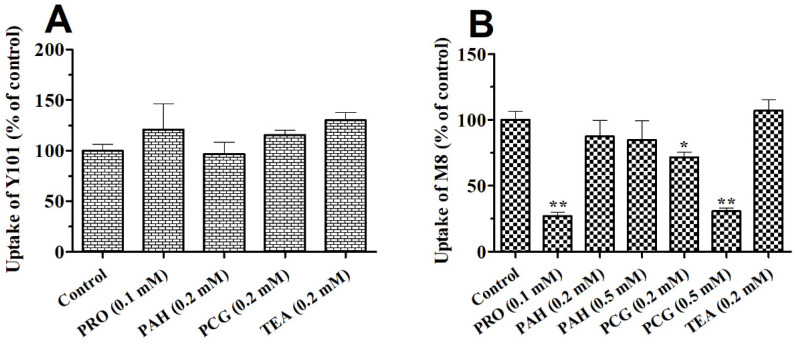
Effects of OATs inhibitors and substrates on the uptake of Y101 (2.0 µM, (**A**)) and M8 (5.0 µM, (**B**)) in kidney slices (*, *p* ˂ 0.05; **, *p* ˂ 0.01 vs. control). Data are expressed as mean ± SD, *n* = 3.

To define the mechanism of the renal excretion of Y101 and its metabolites, an in vitro uptake assay using fresh rat kidney slices was performed. PRO was selected as an OAT1/3 inhibitor and co-incubated with Y101, M8 or M9. Elevated M8 uptake was observed with the increases in incubation time. Furthermore, the uptake of M8 at 37 °C was significantly higher than that at 4 °C (*p* < 0.01, Figure 6B), indicating that the uptake process was temperature-dependent. Unlike M8, the uptake of M9 did not show significant time or temperature dependence (*p* > 0.05, Figure 6C). In combination with the observed minor contribution of M9 to the renal excretion of Y101 in vivo (Figure 2), M9 was ignored in the rest of the experiments. The uptake of Y101 was time- and temperature-dependent (*p* < 0.05, Figure 6A), but at a low level. Additionally, the uptake of M8 was also detected in the kidney slices following the incubation of Y101. The results indicated that the uptake of the M8 was also temperature-dependent and did not decrease in the presence of the PRO (Figure S1). The uptake of the Y101 and M8 increased approximately linearly within the time of 15 min. This led us to select an uptake time of 10 min for the subsequent inhibitory experiments. The uptake of M8 in the kidney slices was inhibited significantly by the OAT3 substrate PCG (0.2 and 0.5 mM), and OAT1/3 inhibitor PRO (0.1 mM), but not by the OAT1 substrate PAH (0.2 and 0.5 mM) or the OCT2 substrate TEA (0.2 mM) (Figure 7B). By contrast, the PAH, PCG, PRO and TEA did not show any influence on the uptake of the Y101 (Figure 7A). These results suggest that the uptake of M8 was partially mediated by Oat3 in the kidneys.

**Figure 8:**
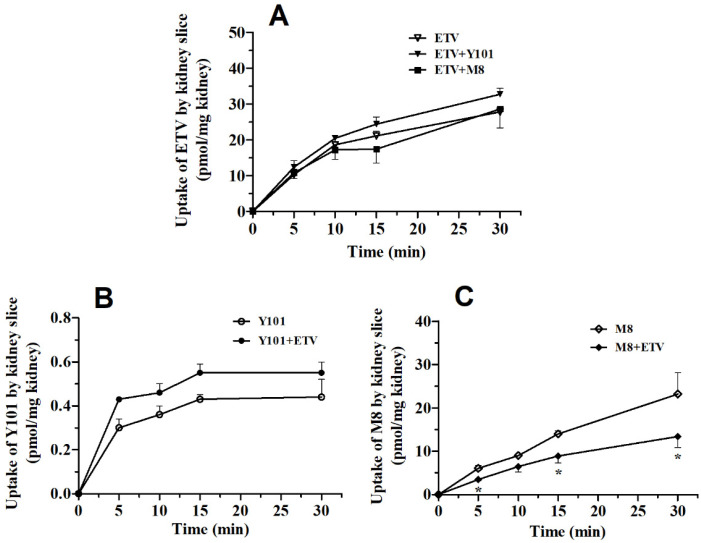
Time-dependent uptake of ETV (10 µM, (**A**)), Y101 (2.0 µM, (**B**)) and M8 (5.0 µM, (**C**)) in kidney slices (*, *p* ˂ 0.05). Data are expressed as mean ± SD, *n* = 3.

To determine the interaction between ETV and Y101 or M8 in vitro, the co-incubation of ETV with Y101 or M8 was performed in the kidney slices. As shown in Figure 8, the observed time-dependent uptake of the ETV was not affected by the presence of Y101 or M8 (Figure 8A). The ETV did not show any inhibitory effects on the Y101 uptake (Figure 8B) but significantly inhibited the uptake of M8 (Figure 8C).

### 2.4. DDI between Y101 and ETV in hOAT1 and hOAT3-HEK293 Cells

**Figure 9:**
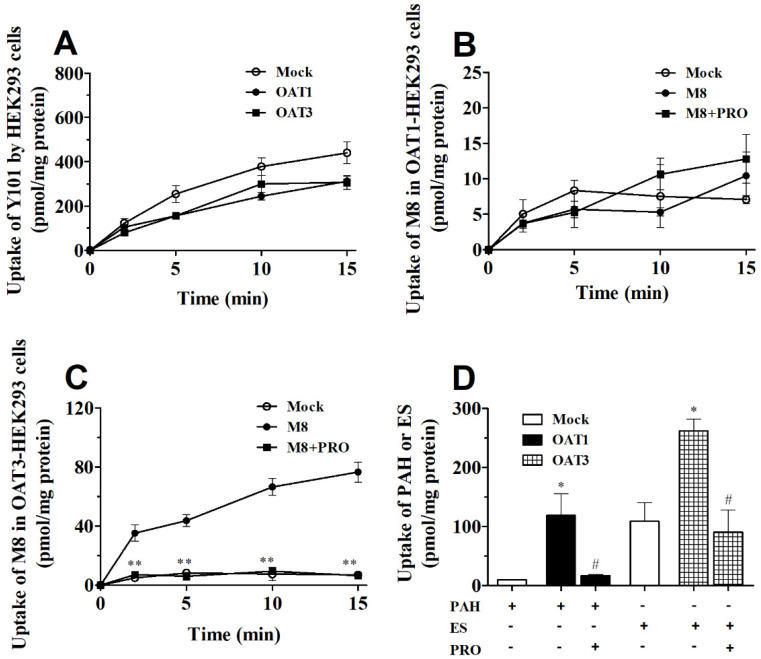
Uptake of Y101, M8, PAH and ES in OAT1/3-HEK293 cells. (**A**): uptake of Y101 (2.0 µM) in mock, hOAT1 and hOAT3-HEK293 cells; (**B**): uptake of M8 (10 µM) in mock and hOAT1-HEK293 cells in the absence or presence of PRO; (**C**): uptake of M8 (10 µM) in mock and hOAT3-HEK293 cells in the absence or presence of PRO (**, *p* ˂ 0.01 vs. M8 alone); (**D**): uptake of PAH (10 µM) and ES (10 µM) with or without PRO (100 µM) for 10 min (*, *p* ˂ 0.05 vs. mock cells; ^#^, *p* ˂ 0.05 vs. without-PRO group). Data are expressed as mean ± SD, *n* = 3.

**Figure 10:**
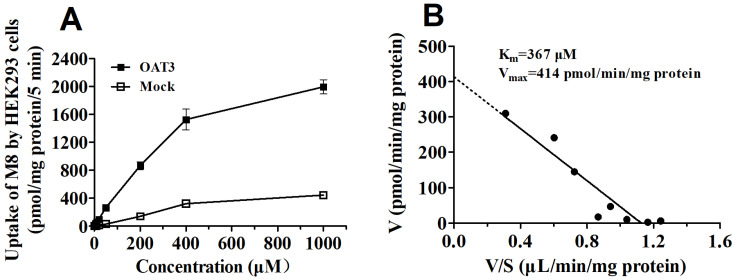
Concentration-dependent profile (**A**) and Eadie–Hofstee plot (**B**) for the uptake of M8 in mock and hOAT3-HEK293 cells. Uptakes of M8 in mock and hOAT3-HEK293 cells were measured at concentrations between 2 and 1000 µM. Data are expressed as mean ± SD, *n* = 3.

Uptake studies using hOAT1 and hOAT3-HEK293 cells were used to verify the involvement of OAT1 and OAT3 in the renal excretion of Y101 and M8. As shown in Figure 9 and Figure 10, uptakes of Y101 at similar rates were observed in the hOAT1, hOAT3-transfected and mock-HEK293 cells (Figure 9A), indicating that Y101 is not a substrate of hOAT1 or hOAT3. The uptake of M8 in the hOAT3-HEK293 cells was significantly higher than that of the mock-HEK293 cells, which was also inhibited by the presence of PRO (Figure 9C), suggesting that M8 is a substrate of hOAT3. The *K*_m_ and *V*_max_ values of M8, calculated by an Eadie–Hofstee plot analysis, were 367 µM and 414 pmol/min/mg protein, respectively (Figure 10). However, similar rates of M8 uptake were found in both hOAT1 and mock-HEK293 cells (Figure 9B), indicating that M8 is not a substrate of hOAT1. In addition, PAH (the probe substrate of OAT1) and ES (the probe substrate of OAT3) were selectively accumulated in hOAT1 and hOAT3-HEK293 cells, respectively, which were inhibited in the presence of PRO (Figure 9D). The findings suggest that the activity of OAT1 and OAT3 in the transfected HEK293 cells was well maintained.

**Figure 11:**
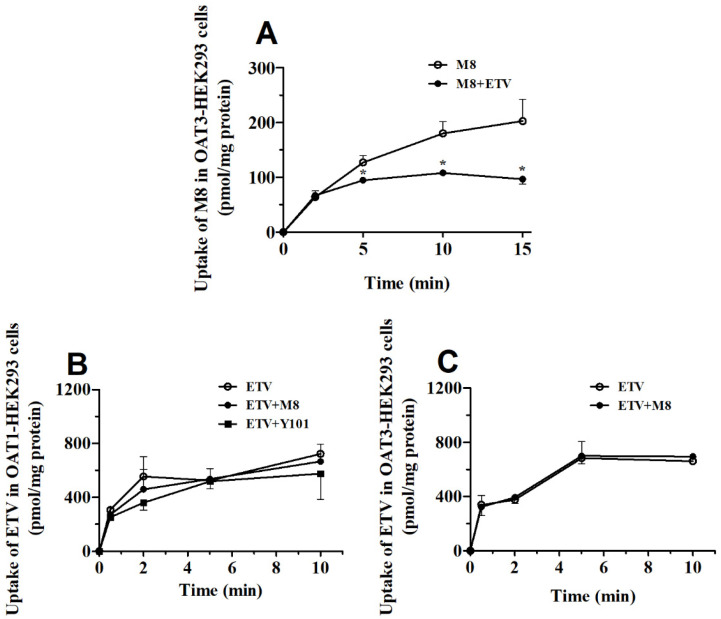
The DDI between Y101 (2.0 µM), M8 (10 µM) and ETV (10 µM) in hOAT1 (**B**) and hOAT3-HEK293 cells (**A**,**C**). *, *p* ˂ 0.05 vs. M8-alone group. Data are expressed as mean ± SD, *n* = 3.

To probe the role of OATs in the DDI between Y101 and ETV in vitro, we examined the uptake interaction of Y101, M8 and ETV in the hOAT1 and hOAT3-HEK293 cells. The uptake of the ETV in the hOAT1 and hOAT3-HEK293 cells was not inhibited by Y101 or M8 (Figure 11B,C). By contrast, the ETV inhibited the uptake of M8 in the hOAT3-HEK293 cells (Figure 11A). The results demonstrated that OAT3 was involved in the DDI between Y101 and ETV through interaction with M8 in vitro.

## 3. Discussion

The novel dipeptide drug candidate Y101 may be applied in combination with other antivirals, such as ETV, for the treatment of HBV infection. A previous study indicated that the renal excretion of Y101 and its metabolites was an important elimination pathway in healthy Chinese subjects [1]. It has been reported that OATs participate in the renal excretion of dipeptide and peptidomimetic drugs and antivirals (such as ETV and acyclovir) [9,10,15]. Therefore, it is possible that OAT-mediated DDI may occur when Y101 and antivirals are simultaneously administered. In this study, we found the participation of OAT3 in the DDI between Y101 and ETV through interaction with M8 in rats in vitro and in hOAT3-transfected HEK293 cells. These results provided prospective data for the potential prediction of DDI in clinical practice.

Organic anion transporters are involved in the renal disposition of a wide variety of endogenous substances, xenobiotics, and drugs [11,12]. Recent clinical studies indicated that the renal excretion of Y101 and M8 accounted for 2.98% and 27.2% of the dose following an oral administration of Y101 to healthy Chinese subjects [1], indicating that renal excretion is an important elimination pathway of Y101. Therefore, the investigation of renal disposition is critical to elucidate the pharmacokinetic characterization of Y101 in rats and humans. Firstly, the cumulative urine-excretion ratios of Y101, M8 and M9 in the rats were 4.16%, 68.1% and 1.42% of the dose following the intravenous administration of Y101 (Figure 2), respectively. Considering that Y101 is a peptidomimetic drug, together with our findings that the renal excretion of Y101 was the primary elimination route, we hypothesized that OATs were involved in the urinary excretion of Y101 and its metabolites and that the DDI between Y101 and ETV took place in the kidneys.

The competitive inhibition of OATs may result in a decrease in renal excretion and an increase in the exposure of drugs in animals or humans [12]. In this study, PRO, a well-known inhibitor of OATs [19], inhibited the renal excretion of M8 in rats treated with an intravenous dose of Y101, but no such inhibition was observed in the animals for Y101 or M9 (Figure 2). In the pharmacokinetic studies, the co-administration of PRO and Y101 led to increases in the plasma concentration and exposure (AUC) of M8 and M9 in rats (Figure 1 and Table 1). This suggests that M8 and M9 might be transported by organic anion transporters in the process of renal excretion. To clarify this mechanism, an in vitro uptake assay using fresh rat kidney slices was carried out to explore the role of OATs in the excretion of Y101, M8 and M9. The observation of the temperature-dependent uptake of Y101 and M8 and the temperature-independent uptake of M9 in the kidney slices (Figure 6) indicates that the transmembrane transport of Y101 and the M8 was mediated by transporters, while the transport of M9 might have occurred through passive diffusion [17,20]. Together with the unchanged CL_R_ of M9 in the presence of PRO in rats (Table 1), we speculated that the increase in AUC for M9 might result from the increased biotransformation from M8 but not from the inhibition of renal excretion by PRO. In the case of the co-incubation with PRO, the uptake of Y101 and M8 was significantly inhibited by the PRO in the kidney slices (Figure 6), which verified that M8 is a substrate of Oat1/3. The discrepancy between the in vivo and in vitro kidney slice results for Y101 (Figure 2C and Figure 6C) was under investigation. We speculate that the uptake of Y101 was predominant and mediated by passive diffusion (Figure 6A, Figure 7A and Figure S1), and that Y101 was metabolized to M8 by enzymes in the kidneys. Therefore, minor changes in the activities of the enzymes responsible for the metabolism of Y101 could have led to significant changes in the low residue level of Y101 in the kidney slices (Figure 6A). Coupled with the findings of the inhibitory uptake assay in the kidney slices (Figure 7), we could conclude that Oat3 participated in the renal uptake of M8. All these findings suggest that the renal transport of M8 via Oats mainly contributed to renal excretion following the intravenous administration of Y101 to the rats. The inhibition of OATs by the PRO decreased the renal excretion of M8, which caused an increase in its plasma concentration and in the AUC in rats.

It has been reported that multiple transporters including OATs, OCT2, OCTNs, MATEs, MDR1 and MRP2 are involved in the renal excretion of ETV [15,16,17,21]. Furthermore, an early study suggested that OAT3 might play a dominant role in the transport of ETV from the blood into renal epithelial cells relative to OAT1 or OCT2 [16]. These findings led us to determine the participation of OATs in the DDI between Y101 and ETV. Interestingly, no significant differences were found in the plasma concentrations, AUCs or cumulative urinary excretion of ETV, Y101, M8 and M9 regardless of intravenous or oral co-administration of Y101 and ETV in rats (Figure 2, Figure 3, Figure 4 and Figure 5, Table 2 and Table 3). The results suggest that Y101 and ETV can be administered together without the risk of DDI in rats in vivo. Furthermore, we carried out mechanistic studies by in vitro uptake assay using rat kidney slices and hOAT1 and hOAT3-HEK293 cells. We found that the *K*_m_ value of M8 for hOAT3 was 367 µM (Figure 10), which exhibited a markedly lower affinity with OAT3 than that of ETV with hOAT3 (23 µM) in the kidneys [15]. A previous study showed a markedly lower affinity of ETV with OAT1 in comparison with that of adefovir, cidofovir and tenofovir, possibly resulting in its lower risk of cellular accumulation, and thus lower potential for cytotoxicity [21]. From this perspective, the different affinity with OAT3 could explained, at least in part, the fact that the M8 did not exert any influence on the uptake of the ETV in the rat kidney slices or the OAT3-HEK293 cells (Figure 8A and Figure 11C). Additionally, we found that the ETV inhibited the uptake of the M8 in the rat kidney slices and the OAT3-HEK293 cells (Figure 8C and 11A), suggesting the involvement of M8-mediated OATs in the interaction between ETV and Y101 in rats and humans in vitro. Previous studies demonstrated that the plasma concentrations of ETV were lower than 50 nM following therapeutic doses in patients [14,22], which suggests that the probability of DDIs being caused by ETV may be low [16]. In addition, our results in vivo indicate that the probability of DDIs between the two drugs mediated by OATs may be low. Based on these findings, we speculate that the OAT3-dependent DDI between Y101 and ETV also seems to be unlikely in humans in vivo. The similarity of OAT3 in humans to that of rats is approximately 90% (the gene sequences of OAT3 in humans and in rats were obtained from the Gene Bank) [23]. Considering the species difference in OAT3 between rats and humans, we suggest that ongoing monitoring is needed in trials to determine the DDI in the future if ETV and Y101 are to be used in combination in patients.

In summary, M8, a metabolite of Y101, is a substrate of OAT3. OAT3 mediated the renal excretion of M8, which played an essential role in the renal disposition of Y101 in rats and humans. Apparently, M8 mediated the DDI between Y101 and entecavir in rats and humans in vitro via OAT3. The observation of an absence of DDI in rats allowed us to speculate that it would be safe to use Y101 with ETV, a substrate of OAT3, in clinic. Additional trials are needed in the future if ETV and Y101 are to be administered simultaneously in patients. The results in the present study provide useful information with which to predict the DDIs between Y101 and other drugs that act as substrates of OAT3 in patients.

## 4. Material and Methods

### 4.1. Chemicals and Reagents

The Y101 (with a purity of >99.5%), M8 (with a purity of >99.0%) and M9 (with a purity of >99.0%) were provided by Guizhou Bailing Group Pharmaceutical Co., Ltd. (Anshun, China). Estrone 3-sulfate sodium salt (ES) was purchased from Toronto Research Chemicals (Toronto, ON, Canada). The PRO and ETV were purchased from Raw Material Medicine Reagent Co., Ltd. (Nanjing, China). Tetraethyl ammonium (TEA), PAH and PCG were purchased from Shanghai Aladdin Biochemical Technology Co., Ltd. (Shanghai, China). Pentobarbital sodium salt was purchased from Tianjin Yifang Technology Co., Ltd. (Tianjin, China). Heparin sodium and sodium carboxymethylcellulose were obtained from Beijing Solarbio Science and Technology Co., Ltd. (Beijing, China). All other reagents were of analytical grade and were commercially available.

### 4.2. Animals

Male Sprague–Dawley (SD) rats (7–10 weeks, 180–220 g, SPF grade) were purchased from Beijing Vital River Laboratory Animal Technology Co., Ltd. (license number SCXK (Beijing, China) 2016-0006). Artificial lighting in the animal housing facility was switched on and off at 12 h intervals. The temperature, humidity, and number of air changes in the barrier system were kept at 20 °C to 26 °C, 40% to 70%, and not less than 15 fresh air intakes per hour, respectively. The SD rats were allowed free access to a water and chow diet but were fasted overnight (with water ad libitum) prior to each experiment. The rats for pharmacokinetic experiments were surgically prepared with indwelling jugular cannulas three days prior to administration in an SPF animal room (license number SYXK (Tianjin) 2018-0008). All animal experiments were performed according to local institutional guidelines for the care and use of laboratory animals.

### 4.3. Pharmacokinetic Study in Rats

The effect of PRO on the pharmacokinetics of Y101 was studied in rats. Male rats surgically prepared with indwelling jugular cannula were randomly divided into two groups (*n* = 3): (1) Y101 alone (25 mg/kg) as a control and (2) Y101 + PRO (25 mg/kg for Y101 and 100 mg/kg for PRO) as an experimental group. The intravenous dose of Y101 was prepared as previously described [24]. Both Y101 and PRO were injected by caudal vein to rats at volume of 5 mL/kg.

Additionally, DDI between Y101 and ETV was investigated by monitoring time-course plasma Y101 in rats with surgery as described above. Animals were randomly placed into three groups (*n* = 4): (1) Y101 alone (25 mg/kg for intravenous treatment and 60 mg/kg for oral administration); (2) ETV (0.06 mg/kg); and (3) Y101 (25 mg/kg for intravenous treatment and 60 mg/kg for oral administration) + ETV (0.06 mg/kg). ETV was dissolved in saline at concentration of 12 µg/mL. Rats received an oral or intravenous dose of Y101 and/or ETV at volume of 5 mL/kg. The doses used in in vivo pharmacokinetic studies were selected in accordance with therapeutic doses of Y101 and ETV commonly used clinically and in previous studies [1,15].

Following administration, serial blood samples were collected through jugular vein pre-dose (0 h) and post-dose at 0.033, 0.083, 0.25, 0.5, 1, 1.5, 2, 3, 4, 6, 9, 12, 24 and 30 h in heparinized Eppendorf tubes. The resulting samples were centrifuged at 13,800× *g* at 4 °C for 5 min to obtain plasma. All plasma samples were stored at −70 °C until analysis.

### 4.4. Renal Excretion Study in Rats

To investigate the effects of PRO on the renal excretion of Y101 and its metabolites, rats received an intravenous dose of Y101 (25 mg/kg) or Y101 + PRO (25 mg/kg for Y101 and 100 mg/kg for PRO) via the caudal vein. In order to verify the DDI between Y101 and ETV in renal excretion, rats received an intravenous dose of Y101 (25 mg/kg) and/or ETV (0.06 mg/kg) by caudal vein.

Following administration, urine was collected in metabolic cages (Tecniplast, Buguggiate, Italy) at 0 h (prior to administration) and the following timepoints post dose: 2, 4, 6, 8, 12, 24 and 30 h. Subsequently, urine samples were stored at −70 °C until analysis. The cumulative urinary excretion and renal clearance (CL_R_) were calculated as previously described [25].

### 4.5. In Vitro Uptake Assay in Kidney Slices

The renal cortex was cut into 300 µm slices with a ZQP-86 tissue slicer (Shanghai Zhisun Equipment Co., Ltd., Shanghai, China; surface area 0.15 cm^2^). In brief, rats were anesthetized with pentobarbital sodium salt, and kidneys were quickly removed and placed in Krebs-bicarbonate slicing buffer (120 mM NaCl, 16.2 mM KCl, 1.2 mM MgSO_4_, 1.0 mM CaCl_2_, 10 mM Na_2_HPO_4_/NaH_2_PO_4_, pH 7.4) at 4 °C with 95% O_2_. Next, the kidney slices were prepared as previously described [25]. Two slices per well were placed into 37 °C buffer filled with oxygen for pre-incubation for 3 min and then gently moved into a 24-well cell culture plate containing 1 mL oxygenated buffer at 4 °C or 37 °C. The uptakes of ETV (10 µM), Y101 (2.0 µM), M8 (5.0 µM) and M9 (1.0 µM) were measured at 5, 10, 15 and 30 min after the treatment, respectively. In inhibition assays, several selective inhibitors of transporters including PRO (0.1 mM), PCG (0.2 and 0.5 mM), PAH (0.2 and 0.5 mM) and TEA (0.2 mM) were individually mixed with buffer containing Y101 or M8, and the final concentration of organic solvent did not exceed 1% (*v*/*v*). Following incubation at the designated time, kidney slices were collected, rinsed with ice-cold buffer three times, and dried on filter paper. After weighing and homogenization (IKA-T 10 homogenizer; IKA, Staufen, Germany), the concentrations of Y101, M8, M9 and ETV of kidney slice homogenates were determined as in the description below.

### 4.6. In Vitro Uptake Assay in hOAT1 and hOAT3-HEK293 Cells

The hOAT1-HEK293, hOAT3-HEK293 and mock cells were cultured in Dulbecco modified Eagle medium (DMEM) containing 10% fetal bovine serum (FBS) and 1% penicillin/streptomycin in an atmosphere of 5% CO_2_ air at 37 °C. The HEK293 cells were plated on 24-well plates at a density of 5 × 10^5^ cells/well. Following culturing for 48 h with nearly confluent cells, an uptake assay was performed as previously described [19,20,25]. In brief, cells were washed three times with Hank’s balanced salt solution (HBSS) and pre-incubated in the transporter buffer (containing 118 mM NaCl, 23.8 mM NaHCO_3_, 4.8 mM KCl, 1.0 mM KH_2_PO_4_, 1.2 mM MgSO_4_, 12.5 mM HEPES, 5.0 mM glucose, and 1.5 mM CaCl_2_, pH 7.4) at 37 °C for 15 min. Uptake assay was initiated by adding 1 mL of the transporter buffer containing Y101, M8, ETV or probe substrate. Following incubation with gentle shaking, the uptake was terminated at designated time by removing the transporter buffer and washing cells three times with 1 mL of ice-cold HBSS. Next, substrates of Y101, M8, ETV, PAH and ES in cell monolayer were released by lysing the cells with 0.3 mL of 0.1% Triton X-100 for 2 h and determined by liquid chromatography–tandem mass spectrometry (LC–MS/MS). In the time-course uptake assay, Y101 (2.0 µM) and M8 (10 µM) in the presence or absence of PRO (100 µM) were measured in hOAT1-HEK293, hOAT3-HEK293 and mock cells. The concentration-dependent uptake of M8 was determined in hOAT3-HEK293 cells, and incubation time (5 min) was optimized. The uptake of M8 (10 µM) with or without ETV was examined in hOAT3-HEK293 cells. The uptake of ETV (10 µM) with or without Y101 or M8 was also measured in hOAT1-HEK293 and hOAT3-HEK293 cells, respectively. Furthermore, PAH and ES at concentrations of 10 µM were used as positive control in hOAT1-HEK293 and hOAT3-HEK293 cells, respectively. Protein concentrations were measured by the bicinchoninic acid procedure (Solarbio, Beijing, China) using bovine serum albumin as the standard.

### 4.7. Sample Preparation and LC-MS/MS Analysis

Biological samples were prepared as previously described [15,26]. Aliquots (50 µL) of urine, plasma, kidney homogenates, or cell lysates were individually mixed with 150 µL methanol and 50 µL working solution of internal standard (acetaminophen or bestatin), followed by vortexing for 1 min and centrifuging at 13,800 *g* for 10 min. The resulting supernatant (50 µL) was mixed with 200 µL of 50% (*v*/*v*) methanol-water, vortexed for 1 min, and centrifuged at 13,800× *g* for 5 min. The resulting supernatant (1 µL) was subjected to LC-MS/MS for the assessment of Y101, M8, M9 and ETV, respectively. Acetaminophen (250 ng/mL) and bestatin (1.0 µg/mL) were used as an internal standard for quantifying ETV, Y101, M8 and M9 in biological samples, respectively. The concentrations of ETV, Y101, M8 and M9 were determined by an AB QTRAP 5500 LC–MS/MS System (Foster City, CA, USA) as previously described [26]. Chromatographic separation was achieved on an Infinitylab Poroshell 120 EC-C_18_ (50 mm × 2.1 mm, 2.7 µm, Agilent Technology Inc., CA, USA) at 40 °C. The mobile phase consisted of formic acid-acetonitrile (0.05:100, *v*/*v*) and a mixture of 1 mmol-ammonium acetate-acetonitrile-formic acid (95:5:0.05, *v*/*v*/*v*) at a flow rate of 0.3 mL/min. Mass spectrometry was performed in multiple reactions monitoring (MRM) mode at the specific ion transitions of *m*/*z* 490.2→339.4 for Y101, *m*/*z* 357.2→105.0 for M8, *m*/*z* 373.2→105.0 for M9, *m*/*z* 278.1→152.0 for ETV, 152.0→110.0 for acetaminophen and *m*/*z* 309.2→120.1 for bestatin [15,26]. Analyst1.5.2 software was used for data processing and analysis.

### 4.8. Data Analysis

Non-compartmental analysis with Phenix WinNonlin (version 8.1; Pharsight, Certara Corp, Princeton, NJ) was used to calculate the main pharmacokinetic parameters of ETV, Y101, M8 and M9 for individual rats [24]. The observed values were reported for maximum concentration (*C*_max_) and time to reach maximum concentration (*T*_max_), while the area under the plasma concentration–time curve (AUC) was calculated via the default trapezoidal-up, log-trapezoidal-down approach.

The SPSS 13.0 software was employed for all statistical data analysis. GraphPad Prism 8 software (La Jolla, California) was used for drawing the figures. Results are expressed as mean ± standard deviation (SD) and one-way analysis of variance was performed for comparison between various groups. Values of *p* < 0.05 or *p* < 0.01 were statistically significant.

## Figures and Tables

**Table 1:** Pharmacokinetic parameters of Y101, M8 and M9 after intravenous administration of Y101 (25 mg/kg) and Y101 + PRO (100 mg/kg). Data are expressed as mean ± SD (* *p* < 0.05, ** *p* < 0.01 vs. Y101 group; *n* = 3).

Compound	Parameters	Unit	Y101			Y101 + PRO		
Y101	*C*_0_	ng/mL	16,029	±	7069	15,770	±	3818
	AUC_0-t_	ng·h/mL	2838	±	481	2018	±	173
	AUC_0-∞_	ng·h/mL	2855	±	488	2031	±	173
	*V*_d_	L/kg	10.2	±	1.22	12.5	±	3.74
	*t*_1/2_	h	0.798	±	0.119	0.706	±	0.227
	CL_p_	L/h/kg	8.94	±	1.65	12.4 *	±	1.01
	CL_R_	L/h/kg	0.366	±	0.0742	0.317	±	0.0463
M8	C_max_	ng/mL	3887	±	567	10,133 **	±	702
	*T*_max_	h	0.667	±	0.289	0.500	±	0
	AUC_0-t_	ng·h/mL	8299	±	278	21,865 **	±	620
	AUC_0-∞_	ng·h/mL	8356	±	274	21,919 **	±	626
	*t*_1/2_	h	4.07	±	1.11	3.45	±	0.955
	CL_R_	L/h/kg	6.00	±	0.614	4.96 *	±	0.265
M9	C_max_	ng/mL	156	±	27.7	219 *	±	16.5
	*T*_max_	h	1.17	±	0.289	1.33	±	0.289
	AUC_0-t_	ng·h/mL	335	±	42.3	527 **	±	21.4
	AUC_0-∞_	ng·h/mL	343	±	44.9	536 **	±	15.2
	*t*_1/2_	h	1.01	±	0.143	1.32	±	0.414
	CL_R_	L/h/kg	0.125	±	0.00356	0.120	±	0.00963

**Table 2:** Pharmacokinetic parameters of ETV, Y101, M8 and M9 after intravenous administration of Y101 (25 mg/kg) and Y101 + ETV (0.06 mg/kg). Data are expressed as mean ± SD (* *p* < 0.05 vs. Y101 group; *n* = 4).

Compound	Parameters	Unit	ETV			Y101 + ETV		
ETV	C_0_	ng/mL	26.4	±	1.63	19.1	±	10.0
	AUC_0-t_	ng·h/mL	9.21	±	2.07	6.31	±	1.14
	AUC_0-∞_	ng·h/mL	9.79	±	2.41	6.76	±	1.24
	*V*_d_	L/kg	11.7	±	3.19	16.4	±	4.33
	*t*_1/2_	h	1.28	±	0.233	1.28	±	0.393
	CL_p_	L/h/kg	6.41	±	1.53	9.09 *	±	1.52
	CL_R_	L/h/kg	2.17	±	0.101	2.00	±	0.347
Compound	Parameters	Unit	Y101			Y101 + ETV		
Y101	C_0_	ng/mL	10,640	±	1350	6281	±	3110
	AUC_0-t_	ng·h/mL	2353	±	272	1674	±	462
	AUC_0-∞_	ng·h/mL	2367	±	269	1692	±	456
	*V*_d_	L/kg	10.6	±	2.89	15.5	±	7.55
	*t*_1/2_	h	0.682	±	0.146	0.660	±	0.187
	CL_p_	L/h/kg	10.7	±	1.15	15.5	±	3.76
	CL_R_	L/h/kg	0.431	±	0.0921	0.476	±	0.0788
M8	C_max_	ng/mL	2665	±	524	2520	±	363
	*T*_max_	h	0.500	±	0.354	0.438	±	0.125
	AUC_0-t_	ng·h/mL	5828	±	726	6096	±	726
	AUC_0-∞_	ng·h/mL	5862	±	735	6146	±	742
	*t*_1/2_	h	1.93	±	0.378	2.65 *	±	0.384
	CL_R_	L/h/kg	6.90	±	0.719	6.35	±	0.614
M9	C_max_	ng/mL	134	±	27.9	171	±	55.1
	*T*_max_	h	1.13	±	0.250	1.25	±	0.289
	AUC_0-t_	ng·h/mL	547	±	196	464	±	168
	AUC_0-∞_	ng·h/mL	553	±	196	471	±	168
	*t*_1/2_	h	1.14	±	0.143	1.80 *	±	0.282
	CL_R_	L/h/kg	0.148	±	0.00798	0.160	±	0.0246

**Table 3:** Pharmacokinetic parameters of ETV, Y101, M8 and M9 after oral administration of Y101 (60 mg/kg) and Y101 + ETV (0.06 mg/kg). Data are expressed as mean ± SD (*n* = 4).

Compound	Parameters	Unit	ETV			ETV + Y101		
ETV	C_max_	ng/mL	5.54	±	2.08	2.94	±	0.351
	*T*_max_	h	0.5	±	0	0.875	±	0.250
	AUC_0-t_	ng·h/mL	8.15	±	1.97	6.76	±	0.941
	AUC_0-∞_	ng·h/mL	8.49	±	1.98	7.41	±	1.33
	*t*_1/2_	h	1.10	±	0.392	1.45	±	0.334
Compound	Parameters	Unit	Y101			Y101 + ETV		
Y101	C_max_	ng/mL	668	±	392	843	±	418
	*T*_max_	h	0.250	±	0	0.313	±	0.125
	AUC_0-t_	ng·h/mL	1574	±	394	1617	±	164
	AUC_0-∞_	ng·h/mL	1622	±	368	1681	±	183
	*t*_1/2_	h	0.998	±	0.291	1.11	±	0.261
M8	C_max_	ng/mL	2033	±	536	1983	±	563
	*T*_max_	h	2.25	±	0.957	1.50	±	1.00
	AUC_0-t_	ng·h/mL	10,129	±	731	10,339	±	572
	AUC_0-∞_	ng·h/mL	10,203	±	709	10,385	±	572
	*t*_1/2_	h	3.44	±	1.52	3.64	±	0.521
M9	C_max_	ng/mL	144	±	59.1	127	±	50.1
	*T*_max_	h	3.00	±	0.816	2.50	±	1.73
	AUC_0-t_	ng·h/mL	641	±	148	645	±	120
	AUC_0-∞_	ng·h/mL	645	±	148	657	±	122
	*t*_1/2_	h	1.16	±	0.171	1.46	±	0.269

## Data Availability

The data presented in this study are available on request from the corresponding author.

## Supplementary Materials

The following supporting information can be downloaded at: https://www.mdpi.com/article/10.3390/molecules28041995/s1, Figure S1: Time-dependent and temperature-dependent uptake of M8 following incubation of Y101 (2.0 µM) in kidney slices.

## Sample Availability

Samples of the compounds are available from the authors.

## Abbreviations

ACEI, angiotensin-converting enzyme inhibitor; AUC, area under the plasma concentration–time curve; Y101, bentysrepinine; *C*_max_, peak concentration; DDI, drug–drug interaction; ETV, entecavir; ES, estrone-3-sulfate; HBV, hepatitis B virus; hOAT, human OAT; LC-MS/MS, liquid chromatography coupled with tandem mass spectrometry; MRM, multiple reactions monitoring; OAT, organic anion transporter; OCT, organic cation transporter; PAH, *p*-aminophenol acid; CL_p_, plasma clearance; CL_R_, renal clearance; PRO, probenecid; *t*_1/2_, elimination half-life; *T*_max_, the time of peak concentration.
