# Supplementary material for: OAT3 Participates in Drug–Drug Interaction between Bentysrepinine and Entecavir through Interactions with M8—A Metabolite of Bentysrepinine—In Rats and Humans In Vitro
Source: Molecules. 2023 Feb 20;28(4):1995. doi: 10.3390/molecules28041995 (PMC9967645; doi:10.3390/molecules28041995)
Supplement: Supplementary file 1 [file molecules-28-01995-s001.zip › molecules-2005445-supplementary.pdf]

# Supplementary materials: OAT3 Participates in Drug-Drug Interaction between Bentysrepinine and Entecavir through Interactions with M8—A Metabolite of Bentysrepinine—In Rats and Humans In Vitro

Aijie Zhang[†], Fanlong Yang[†], Yang Yuan, Cai Li, Xiaokui Huo, Jing Liu, Shenzhi Zhou, Wei Li, Na Zhang, Jianfeng Liu, Shiqi Dong, Huirong Fan[*], Ying Peng[*], and Jiang Zheng[*]

## Figures:

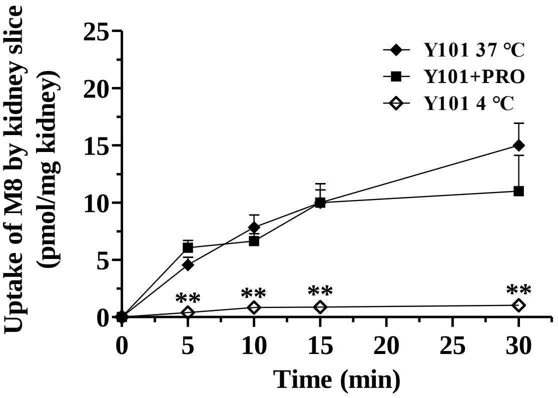


**Figure S1**. Time-dependent and temperature-dependent uptake of M8 following incubation of Y101 (2.0 μM) in kidney slices. The concentration of PRO was 100 μM. (*, $p < 0.05$; **, $p < 0.01$). Data are expressed as mean ± SD, n = 3.
